# Analysis of the Abrasive-Type Influence on the Effectiveness of Rotary Cleaning of Machine Parts with Complex Geometric Features

**DOI:** 10.3390/ma13225144

**Published:** 2020-11-15

**Authors:** Sylwester Korga, Kamil Żyła, Jerzy Józwik

**Affiliations:** 1 Department of Computer Science, Faculty of Electrical Engineering and Computer Science, Lublin University of Technology, Nadbystrzycka 36B, 20-618 Lublin, Poland; k.zyla@pollub.pl; 2 Department of Production Engineering, Faculty of Mechanical Engineering, Lublin University of Technology, Nadbystrzycka 36, 20-618 Lublin, Poland; j.jozwik@pollub.pl

**Keywords:** machine parts corrosion, mechanical cleaning, abrasives, equipment regeneration, computer-aided analysis

## Abstract

This work presents the analysis of functional relationships between fraction size of abrasives and geometric parameters of surfaces after rotary cleaning. The influence of an abrasive type on the effectiveness of rotary cleaning of machine parts with complex geometric features was determined as well. The process of mechanical cleaning, using a rotational method, of clutch springs was performed in the proprietary device for rotational cleaning, which was followed by the computer-aided analysis of the obtained results. The research process was carried out using abrasive materials such as grinding stone, sand, basalt, glass, and fine gravel, and the test samples were clutch springs after eight years of operation. Based on calculated three-dimensional (3D) roughness values of the cleaned samples’ surfaces, qualitative classification of abrasives was determined. The most effective material turned out to be fine gravel, while the worst results were related to basalt usage.

## 1. Introduction

The phenomenon of corrosion affects many groups of materials, nevertheless the most often it is associated with is the destruction of metals. The corrosion could be simply explained as the process of material degradation caused by the factors of the surrounding environment. Very good physical and chemical properties of steel are the reason for its use in many branches of industry. One of the most significant problems in construction and exploitation of machines is the corrosive destruction of iron alloys [1,2,3,4,5]. The effects of this process are losses—direct ones, related to the costs of damaged materials and construction protection, as well as indirect ones, related to the operation of machines. Contemporary research on corrosion tends to multi-directionality, becoming increasingly complex and expensive [6,7,8,9,10]. The low corrosion resistance of construction materials is an obstacle in various fields of machine development, which is why it is so important to invest in research contributing to the theoretical and practical knowledge about the sources of corrosion and the means of minimizing it [11,12,13].

The economic effects of corrosion are related to losses caused by this phenomenon and expenditures on corrosion prevention. Literature studies revealed that corrosion is a natural process which is amplified by the increase of environmental pollution [14,15,16,17]. Machine parts being subject to corrosion should in many cases be protected and in extreme cases, they may be subject to regeneration [18,19,20,21,22,23]. This involves specialized devices and technologies based on mechanical and chemical phenomena. One of the problems of modern research regarding metal corrosion processes is the selection of appropriate technologies that enable regeneration of machine parts. This means the need to select appropriate workshop equipment to remove corrosion centers. In this way, it is possible to stop the progressing process of destruction and prepare the top layer for the application of protective coatings [24,25,26,27]. High financial outlays, required to protect metals against corrosion, are the result of a number of activities thanks to which corrosion processes are stopped or, to a significant extent, delayed. References [28,29,30] present the problem of cleaning machine parts and methods of their protection. At last, the sources of corrosion processes and corrosion prevention methods were evaluated in References [31,32,33,34].

It is especially troublesome and expensive to remove corrosion centers from surfaces of machine parts with unusual and complex shapes. Therefore, there is a need to develop methods and machines enabling this process to be carried out taking into account low costs. Keeping in mind the abovementioned factors, a special stand for the purpose of corrosion products’ removal using the rotational method was built. In order to meet low-cost requirement as well, the stand was assembled using easily available components, and it was adapted to the use of commonly available abrasives. Car clutch springs were selected as the subject to be cleaned during the research process. In order to assess the cleaning results, reference was made to basic indicators, such as visual assessment and sample weight loss, as well as to advanced computer methods.

The main gains of this work are the analysis of functional relationships between fraction size of abrasives and geometric parameters of surfaces after rotary cleaning using the abovementioned stand, as well as the assessment of the abrasive-type influence on the effectiveness of rotary cleaning of machine parts with complex geometric features. Research described in the following chapters might be especially useful in the context of semi-professional applications, e.g., renovation of mechanical parts that are hardly accessible on the market.

## 2. Materials and Methods

Analyses concerning corrosion phenomenon cover a wide range of research processes in the field of materials science, which are carried out to determine the type of corrosion damage and the causes of corrosion. In the literature [2,4,17,35], corrosion tests are classified according to various criteria, among them according to the type of conditions in which the tests are performed (i.e., tests in conditions of natural environment and in laboratory conditions). Tests in natural conditions are carried out in corrosive environments such as air, soil, or liquids. Operational tests are tests of metal components and machine parts under their operating conditions.

Due to the specificity of the corrosion products’ removal process, manual-mechanical cleaning and mechanical cleaning are distinguished [33,36]. The mechanical method is one of the oldest ways to clean machinery and equipment components. The method is based on a direct action on a corroded surface of an element and removal of corrosion products from its surface, which is discussed in detail in References [28,37]. The mechanical method allows for quick cleaning of components, although it requires the use of mechanized tools. The basic kinds of activities that can be applied during a mechanical cleaning process include hammering, scraping, brushing, needling, grinding, polishing, blast cleaning, and cleaning with loose abrasive, which was characterized in References [10,13,29,37].

The research described in this work followed guidelines and good practices prescribed in the literature for operational tests in the natural environment conditions, as well as for the mechanical method of removing corrosion products with loose abrasive.

### 2.1. Description of the Test Stand Used for Rotational Cleaning

In order to carry out the research process, a test stand was specifically designed and constructed keeping in mind the criterion of cost, availability, and purpose. Due to the mentioned criteria, as well as the purpose and specificity of cleaning machine parts with complex geometric features, it was decided to use a rotational method, where cleaning is carried out using loose abrasive. During the test stand development, construction and technological parameters recommended in the literature were taken into account [1,38,39,40]. The test stand has a unified modular structure, which allows it to be commercialized at low financial outlays. Despite the uncomplicated construction, it can even be used in industrial processes, like the preparation of material for applying paint coatings.

**Figure 1:**
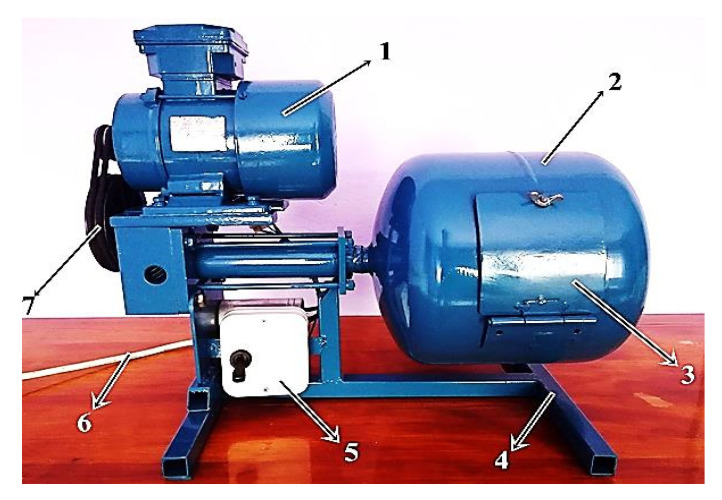
“Corg ver. 1” device for mechanical cleaning, using the rotational method, of machine parts with complex geometric features: 1—engine, 2—rotary drum, 3—drum flap, 4—frame, 5—on/off switch, 6—power cable, 7—belt transmission.

The core part of the test stand is the device for mechanical cleaning of machine parts with complex geometric features using the rotational method. From now on, it will also be called “Corg ver. 1”. The device (see Figure 1) consists of the following components: 1—engine, 2—rotary drum, 3—drum flap, 4—frame, 5—on/off switch, 6—power cable, 7—belt transmission. Its principle of operation is to make the drum rotate by means of the motor drive.

During the cleaning process in “Corg ver. 1”, abrasive and an element to be cleaned cooperate in the horizontal axis of the rotary drum. Oxidized particles of material, that are a product of chemical reactions under environmental factors, are mechanically removed by the abrasive. Such a cleaning process is long-lasting (up to several dozen minutes), mainly due to the small contact surface of the object to be cleaned with the abrasive. Nevertheless, according to the literature [6,25,31], such methods are used with positive effects during machines’ regeneration.

### 2.2. Description of the Research Samples

**Figure 2:**
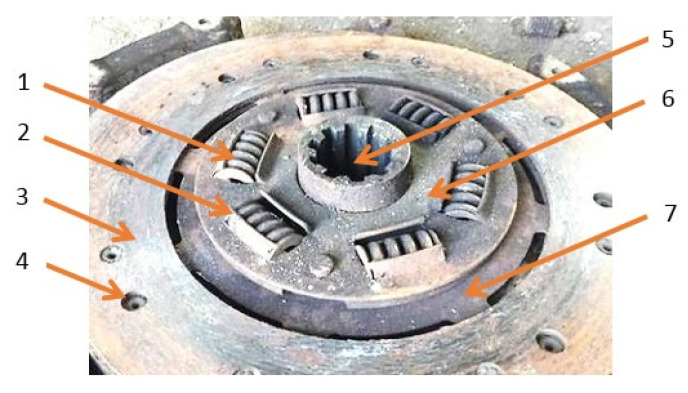
Disk clutch of an FS Żuk delivery car: 1—spring, 2—spring holder, 3—friction surface, 4—rivet, 5—hub, 6—hub disk, and 7—inner disk.

**Figure 3:**
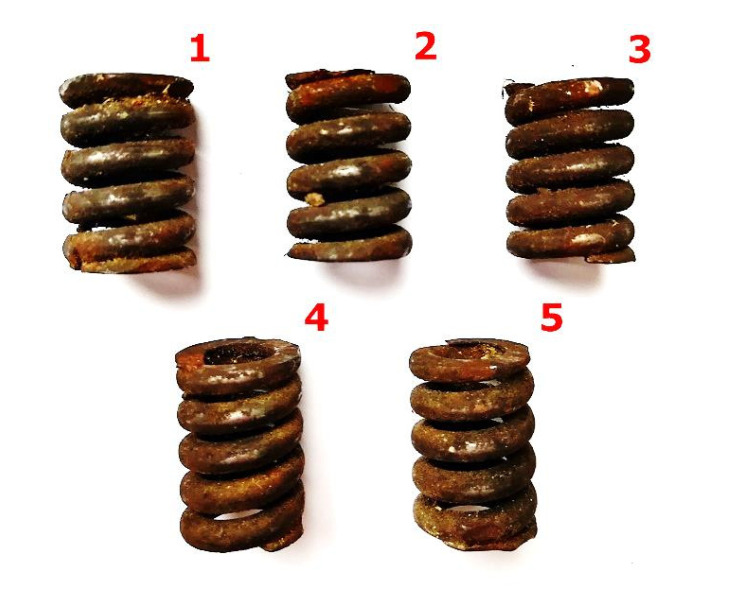
Representative group of 5 spring samples before the cleaning process using: 1—grinding stone, 2—sand, 3—crushed basalt, 4—fragmented glass, 5—fine gravel.

Springs from a car clutch, as they meet characteristics of machine parts of complicated geometric features, were used to verify the design assumptions of the developed device for rotary cleaning, and to choose the most suitable abrasive. Corrosion products removal tests were carried out on five groups of samples, being springs constituting the clutch disc of the FS Żuk delivery car (see Figure 2). Each group contained five springs (1) taken from the clutch disc (6) of catalog number 20-1601130-12. The springs were made of 65G alloy steel, and they were characterized by the following geometric parameters: length 26.5 mm, width 18.5 mm, spring wire thickness 4 mm, and pitch 5.8 mm. They came from clutches of the same type/construction/material. Twenty-five samples with the similar degree of surface oxidation were used in total. The samples worked in changing environmental conditions, during ongoing car exploitation, being exposed to humidity, lubricants, and chemical agents. Figure 3 shows the surface condition of a representative sample from each group before the start of the research process.

**Figure 4:**
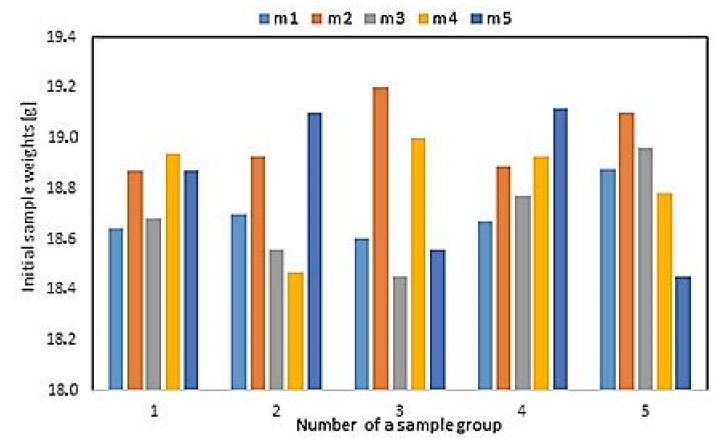
Weight distribution among investigated sample groups.

Spring samples were divided into 5 groups, and each group consisted of 5 springs. Before the cleaning process, each sample was weighted. Figure 4 shows their weight distribution (m1–5 means the number of a sample in a group), for exact values see Table 1. The smallest weight of a sample was 18.45 g and the highest one was 19.20 g. The average sample weights for the groups 1 to 5 were: 18.70 g, 19.00 g, 18.68 g, 18.82 g, and 18.82 g. Measure of data dispersion (variance) for the groups 1 to 5 were: 0.01, 0.06, 0.10, 0.05, and 0.05. The smallest dispersion of weight was registered for the first sample group and the largest one was registered for the third group. The samples were of a similar weight. Difference between the weight values did not exceed 2.7%, therefore they were considered to show weight repeatability.

### 2.3. Description of the Research Procedure

Five groups of test samples were subjected to the cleaning process in the “Corg ver. 1” device. They were cleaned using the following materials: 1—crushed grinding stone, 2—sand, 3—crushed basalt, 4—fragmented glass, and 5—fine gravel. The volume of the drum constituting the cleaning chamber was 7200 cm^3^. 2400 cm^3^ of abrasive was poured into the drum, followed by a group of 5 spring samples. Volume of 2400 cm^3^ was enough to fulfill the requirement of samples being surrounded by each abrasive used. The rotation speed of the cleaning chamber was set to 81 rpm with the engine power of 0.12 kW. After 1 h, the operation was repeated on another type of the abrasive and another group of 5 spring samples. 1 h was the amount of time needed to clean each sample group taking into account the abovementioned conditions and settings.

The surface condition of cleaned samples was inspected and evaluated taking into account the following material properties: surface appearance, degree of surface cleanliness, and the presence of lubricants. It was made on the basis of PN-ISO 8501-1 group of standards [40]. Next, a computer analysis was made. Thanks to the three-dimensional (3D) imaging techniques, a set of high-resolution surface topography slices was produced, and then the surface features were measured in accordance with the PN-ISO 8501-1 standards. Inspection of samples surface was assisted by the Alicona Infinite Focus G5 system (G5, Bruker Alicona, Graz, Austria).

## 3. Results and Discussion

The first stage of evaluation of the cleaned spring samples was weight and visual assessment. Removal of corrosion products using the rotational method always leads to the sample weight loss. The smallest losses were observed in case of abrasives like basalt and glass. Such effect might be explained by the smaller contact surface between an abrasive and a cleaned sample. See Table 2 for details concerning weight loss in groups of samples.

**Figure 5:**
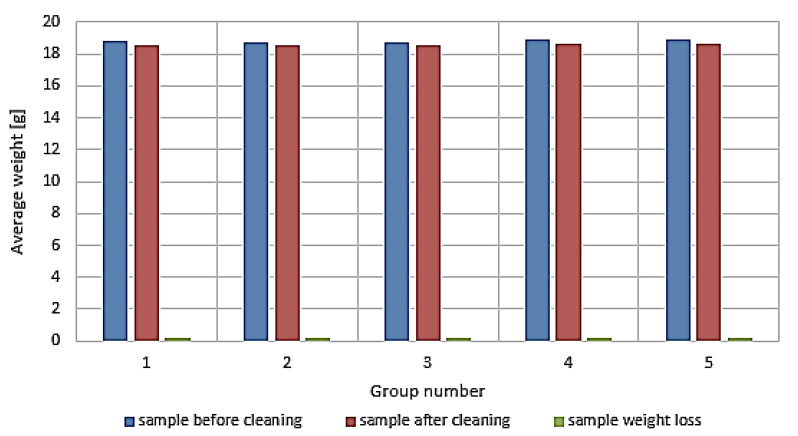
Average sample weight and average weight loss within the sample groups.

Based on the samples’ weight loss distribution after the cleaning process, standard deviation was calculated. It revealed that weights of samples are very similar. Low values of the standard deviation indicate good repeatability around the average value. The summary of samples’ weight is shown in Figure 5.

**Figure 6:**
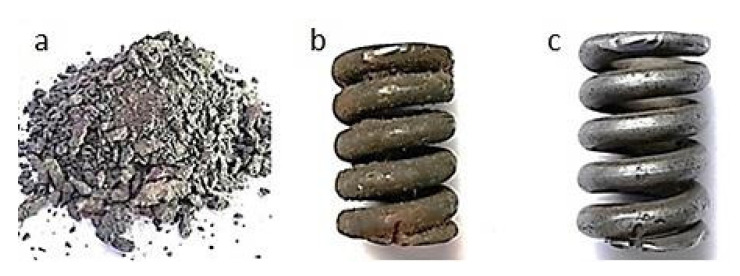
Cleaning using crushed grinding stone: (**a**) view of the abrasive, (**b**) view of a spring sample surface before the cleaning, and (**c**) view of the same spring sample surface after the cleaning.

**Figure 7:**
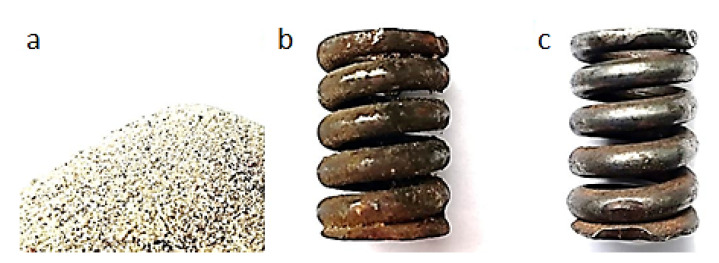
Cleaning using sand: (**a**) view of the abrasive, (**b**) view of a spring sample surface before the cleaning, and (**c**) view of the same spring sample surface after the cleaning.

**Figure 8:**
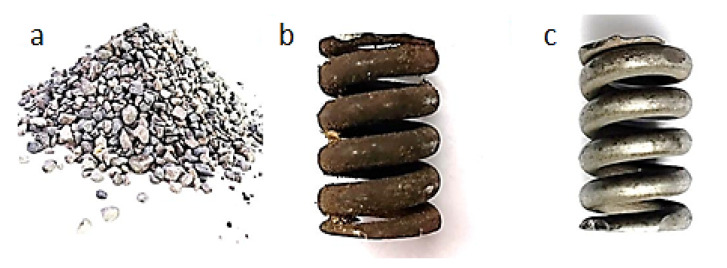
Cleaning using crushed basalt: (**a**) view of the abrasive, (**b**) view of a spring sample surface before the cleaning, and (**c**) view of the same spring sample surface after the cleaning.

**Figure 9:**
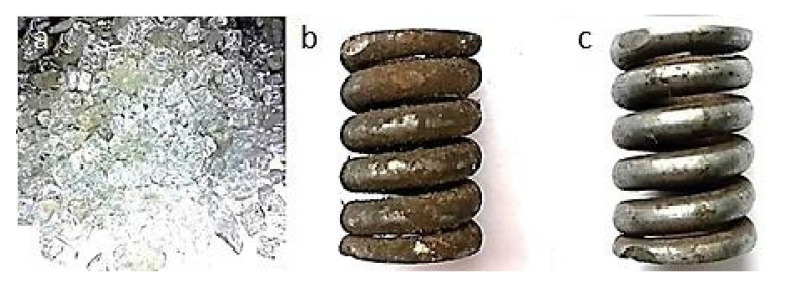
Cleaning using fragmented glass: (**a**) view of the abrasive, (**b**) view of a spring sample surface before the cleaning, and (**c**) view of the same spring sample surface after the cleaning.

**Figure 10:**
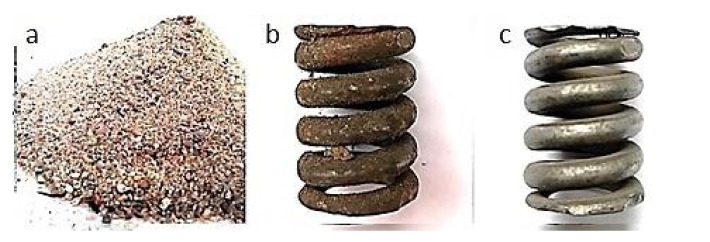
Cleaning using fine gravel: (**a**) view of the abrasive, (**b**) view of a spring sample surface before the cleaning, and (**c**) view of the same spring sample surface after the cleaning.

Figure 6, Figure 7, Figure 8, Figure 9 and Figure 10 show representative spring samples before and after the cleaning process utilizing the following abrasives: grinding stone, sand, basalt, glass, and fine gravel.

**Figure 11:**
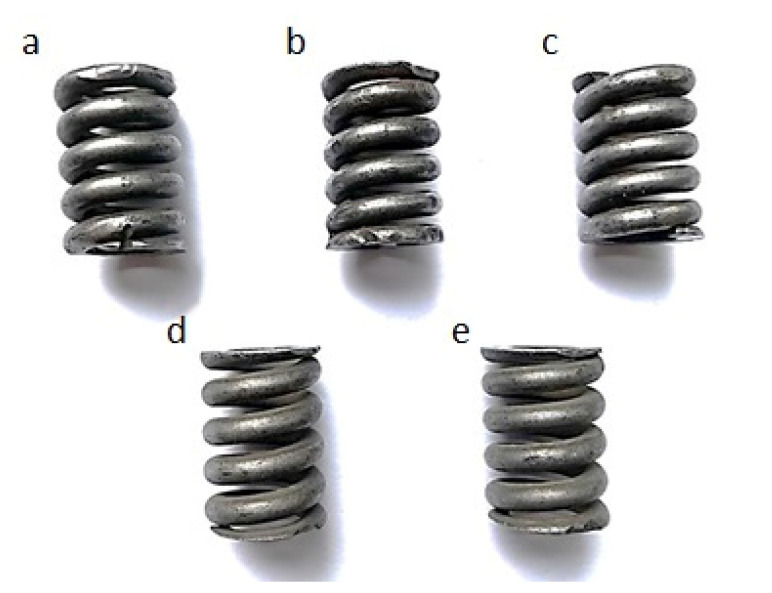
Exemplary spring samples after the cleaning using: (**a**) crushed grinding stone, (**b**) sand, (**c**) crushed basalt, (**d**) fragmented glass, and (**e**) fine gravel.

The visual analysis of samples’ surfaces after the cleaning revealed the presence of insignificant remnants of corrosion products in the surface recesses and asymmetrically distributed damages of the top layer (see Figure 11). All samples were free of lubricants. Samples treated with grinding stone, sand, and basalt had a pronounced gloss. Obtained surfaces differed among themselves depending on the abrasive used. After cleaning a surface with crushed grinding stone, it became almost completely free of corrosion products. The surface of spring marked as (a) and (b) became shiny after cleaning, the surface of spring marked as (c) partly shiny, and in case of springs marked as (d) and (e), it became matt, which can be observed in Figure 11.

**Figure 12:**
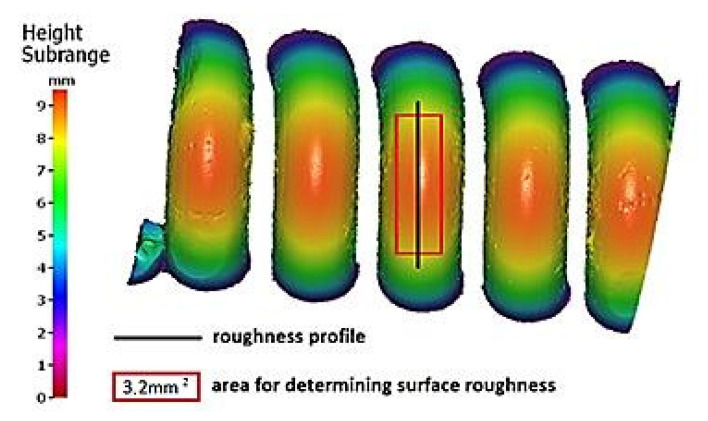
Indication of roughness measurement zones on spring samples after the cleaning process.

According to the literature [6,21,24], the abovementioned assessment of the corrosion degree can be perceived as subjective, thus it is desirable to search for a better solution. This is why the cleaned spring samples were the subject of further analyses. After literature research on computer-aided methods and imaging techniques in corrosion assessment, it was decided to utilize selected 3D surface roughness parameters as a supplementary method of the assessment. In order to obtain the values of these parameters, a non-contact measurement method provided by the Alicona Infinite Focus G5 system was used. Figure 12 shows the area of samples’ surface (3.2 mm^2^), being the subject of further analysis.

**Figure 13:**
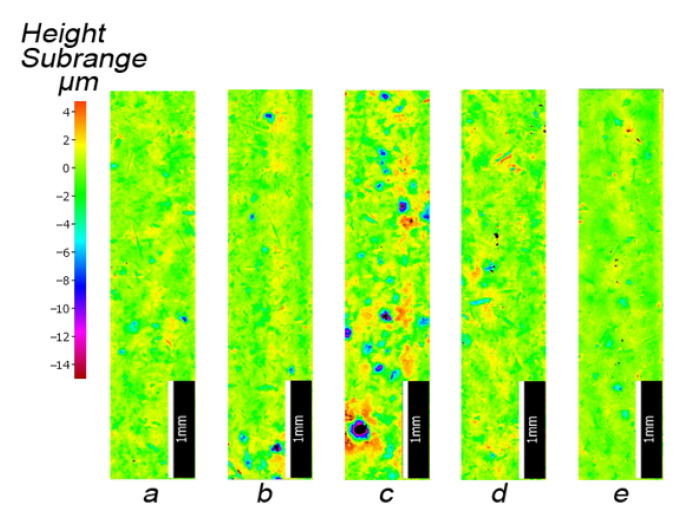
Optical images of geometric structures on surfaces of representative samples cleaned with materials: (**a**) crushed grinding stone, (**b**) sand, (**c**) crushed basalt, (**d**) fragmented glass, and (**e**) fine gravel.

Figure 13 shows images of surface textures generated for spring samples chosen as representative for each sample group cleaned using a particular abrasive. The surface topography maps show typical pits, grooves, craters, and traces of exploitation, which are a kinematic and geometrical representation of abrasive grains. Accurate surface analyses required consideration of not only these maps, but values of 3D surface roughness parameters as well.

The computer-based analysis of the surface topography gave results in the form of values of 3D surface roughness parameters for the five sample groups. The computed results are summarized in Table 3. Columns called “Group 1–5” present average values of these parameters, which were based on values computed for each sample in a particular group. Values in the column called “Standard deviation” were counted basing on average values in columns called “Group 1–5”. Values from Table 3 were used during the assessment of abrasives and samples’ surfaces after the cleaning.

During the analysis of the surface topography maps and 3D surface roughness parameters from Table 3, it was observed that differences in values of *Sa* and *Sq* parameters, computed for research samples, range from several to several dozen percent. Such values of these parameters result from the fraction size of abrasives used for rotational cleaning.

Based on the *Sku* and *Sdq* values, it can be concluded that there is a clear relationship between the obtained sample roughness and the abrasive materials used. Based on the *Sku* kurtosis parameter, it was observed which abrasives give a beneficial effect in the cleaning process. In References [9,30], it is recommended that height parameters should be considered together with kurtosis, because not only the depth of the surface profile recesses but also their shape determines the concentration of stress.

Taking into account the obtained *S10z* roughness parameter values, it can be concluded that the abrasives used for cleaning machine parts can be used to obtain various surface roughness. For surfaces cleaned with sand and basalt, the highest values of roughness parameters *Sdq* and *Sdr* were measured. Tabular data related to these parameters confirms that such surfaces have the highest unevenness and the highest peak density per unit area.

**Figure 14:**
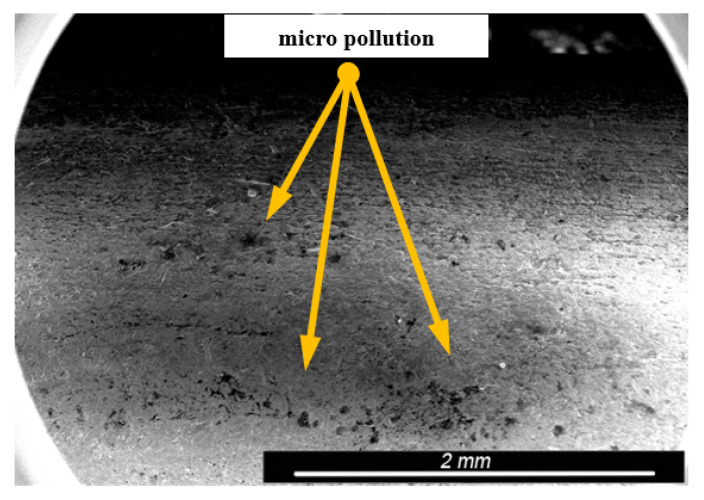
Microscopic image of the sample surface before the exploitation process, magnification of ×55.

**Figure 15:**
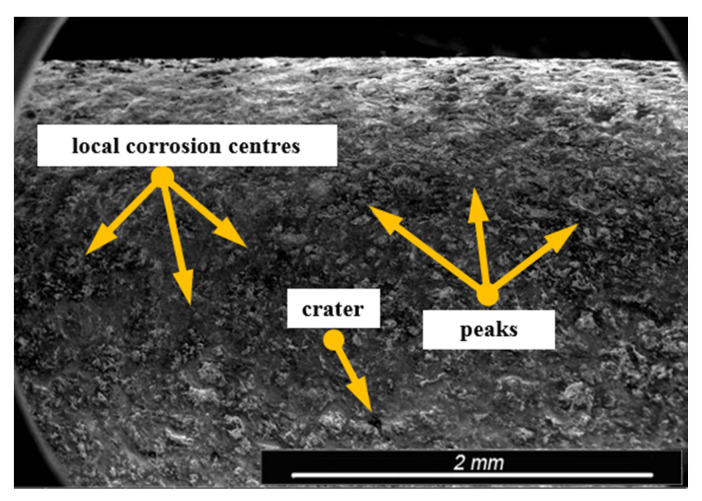
Microscopic image of the sample surface after being subjected to the exploitation process, magnification of ×55.

**Figure 16:**
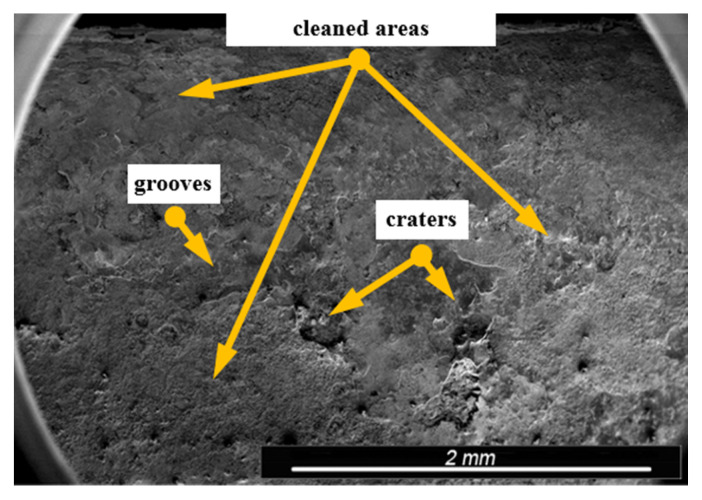
Microscopic image of the sample surface after being cleaned with sand, magnification of ×55.

**Figure 17:**
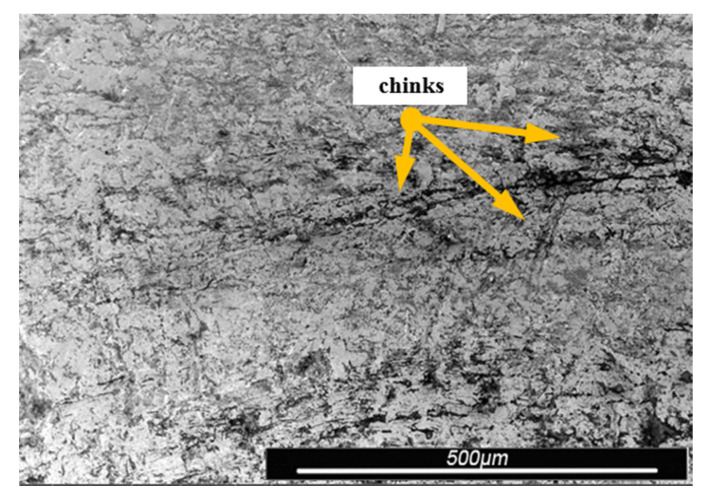
Microscopic image of the sample surface before the exploitation process, magnification of ×200.

**Figure 18:**
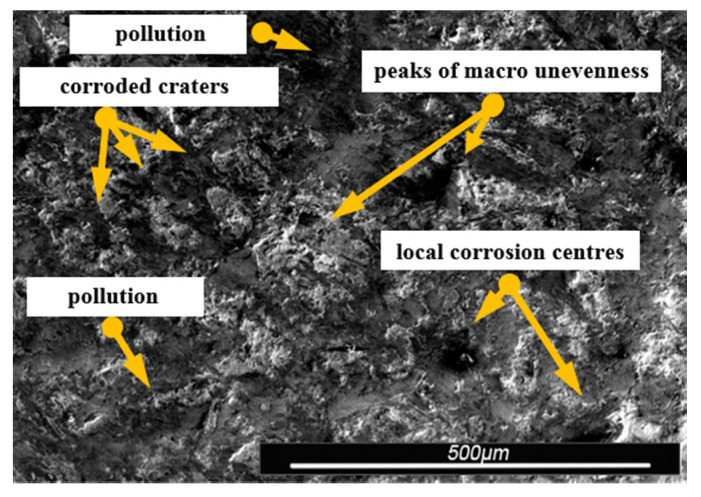
Microscopic image of the sample surface after being subjected to the exploitation process, magnification of ×200.

**Figure 19:**
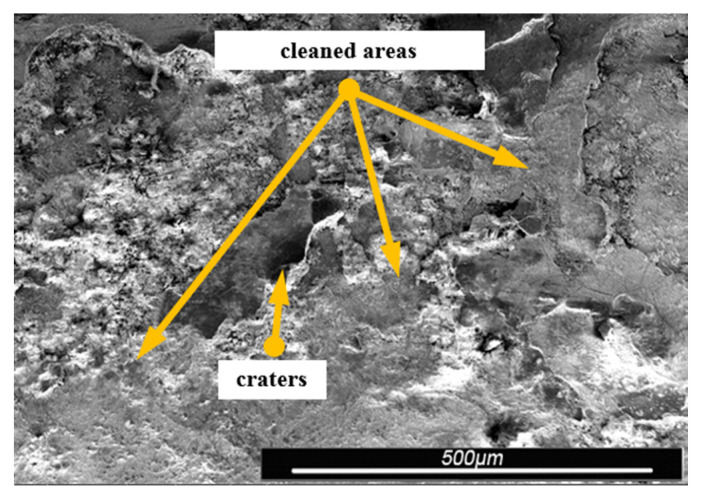
Microscopic image of the sample surface after being cleaned with sand, magnification of ×200.

**Figure 20:**
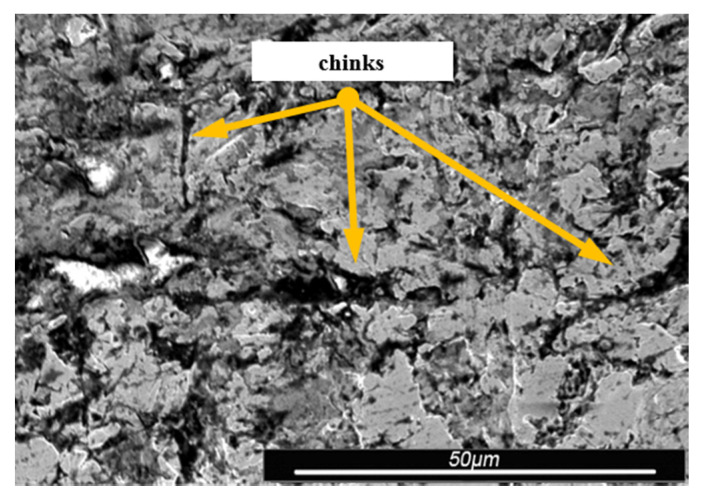
Microscopic image of the sample surface before the exploitation process, magnification of ×2000.

**Figure 21:**
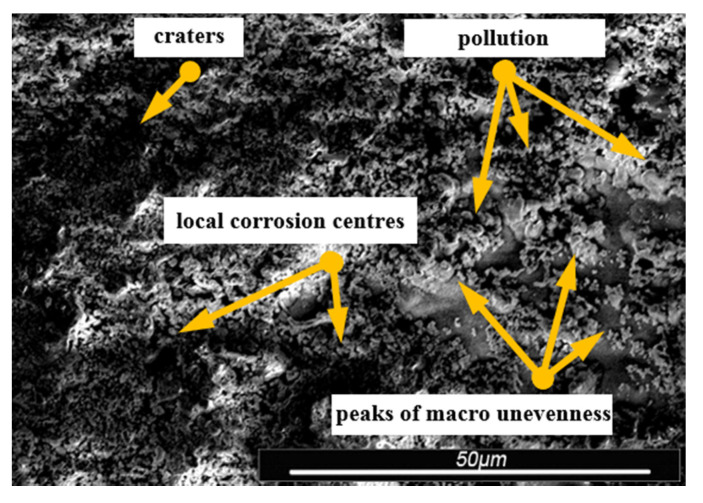
Microscopic image of the sample surface after being subjected to the exploitation process, magnification of ×2000.

**Figure 22:**
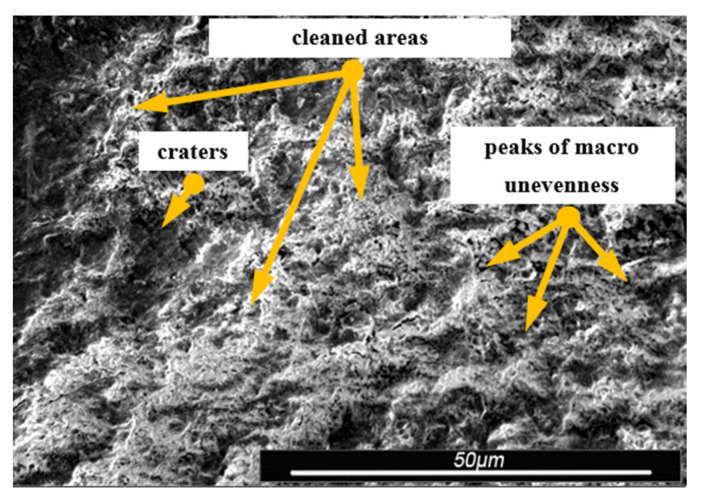
Microscopic image of the sample surface after being cleaned with sand, magnification of ×2000.

Environmental and exploitation factors, that were affecting research samples, caused local damages to the samples’ surfaces as well as developing corrosion micro-cells. In order to assess the correctness/quality of the cleaning processes, metallographic tests of the cleaned samples were performed. For the purpose of taking the metallographic photographs, a Nova Nano SEM 50 device (50 series, FEI, Tokyo, Japan) was used. Exemplary photographs (of the same sample) concerning the process of cleaning with sand are shown in Figure 14, Figure 15, Figure 16, Figure 17, Figure 18, Figure 19, Figure 20, Figure 21 and Figure 22.

Local corrosion centers were observed on the samples’ surfaces before cleaning (seen in Figure 15, Figure 18 and Figure 21). Corrosion affecting the samples is a stress corrosion type, which is especially visible in Figure 16. When observing the samples with ×2000 magnification, no cracks were observed in the outer layer of the material. The absence of macro-cracks indicates that the cleaned samples (springs) can be approved for further use. During the microscopic observation of the samples, no crushing effects were observed, and thus the structure of the object was not changed. Analysis of images presented in Figure 16, Figure 19 and Figure 22, showing the samples cleaned with sand, revealed a diverse structure of the material, including pits, craters, and grooves that were not completely removed. This type of problem also concerned samples cleaned with the remaining abrasives.

During the analysis of the samples, the cyclic operation of the springs was taken into account, which directly affects the speed of the corrosion process. The result of cyclic loading is damage to the top layers and paint coatings. Damage to the continuity of the paint/varnish coating causes pitting. Their nucleation may be the beginning of pitting formation in the macro-scale. It is a process that develops especially in the places of non-metallic inclusions at the grain boundaries.

Another important factor destroying the samples is the stress that occurs in the entire volume of the spring. Fatigue corrosion can be partially prevented by treating the surface layer in a way introducing compressive intrinsic stress or favorable structural changes. Therefore, cleaned springs should be subjected to further securing processes, e.g., thermal, thermo-chemical, or painting processes. The use of such processes will largely eliminate the occurrence of hydrogen corrosion.

Gaps/chinks that occur in the tested samples can be observed on the surface, therefore microscopic examination should be used for the final verification of the suitability of the springs for operation.

**Figure 23:**
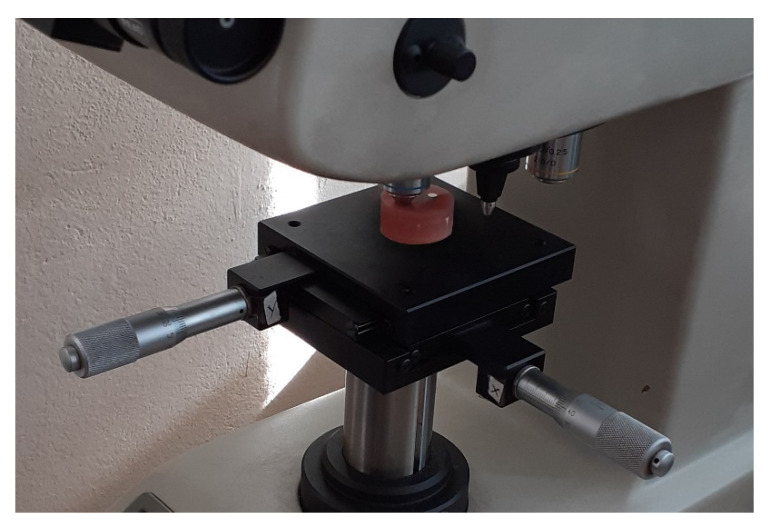
Microhardness examination of spring material.

**Figure 24:**
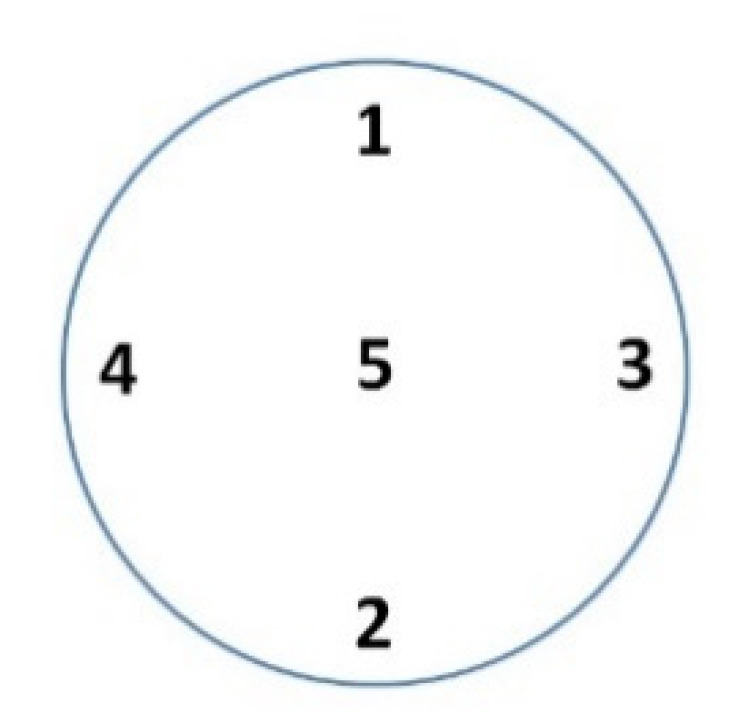
Places in a spring plane where the microhardness measurements were made.

In order to determine the possibility of using abrasives for cleaning the springs, the springs were cut in a plane perpendicular to the axis of symmetry. The Microhardness tester Leco 700AT (LM Series, LECO, San Jose, MI, USA) was used to measure the microhardness of the springs (Figure 23). The measurement was made using the Vickers method with a load of 100 g. Five measurements were made in each measurement plane, as presented in Figure 24. Measurements 1, 2, 3, and 4 were taken radially, while measurement 5 was taken centrally. The summary of results is shown in Table 4.

Based on the data from Table 4, it was assumed that the material microhardness did not increase in the performed process of the samples’ cleaning.

Due to the rotational-abrasive nature of the cleaning process, no effect of directional traces of processing was noticed, which can be important in case of machine parts that work in the tribological environment. After the rotational-abrasive cleaning process, a decrease in the sample roughness value was observed comparing to the samples from before the process. Reducing the roughness value of the samples is a positive factor, because it can reduce the friction coefficient, while in the case of higher roughness values, the friction process can be more intense.

At last, the abovementioned features of cleaned items can be utilized in further machine operation processes. After conducting the research, the qualitative classification of abrasives was developed (see Table 5) on the basis of the results of experimental studies concerning the physical properties of surface profiles.

The surfaces cleaned using the rotary-abrasive method showed similar but still different roughness characteristics of the surface layers. The abrasive that provides the most beneficial surface features was chosen as the best. The authors acknowledge that the results and assumptions described in this work are correct for the abrasives presented in Table 5, that were used during the research process. Using the same abrasives although of different fraction size could provide different results (like qualitative classification of abrasives), which might be perceived as the motivation for further research.

## 4. Conclusions

Mechanical cleaning using the rotational method can be effectively used to remove effects of the corrosion process from machine parts with complex geometric features. After cleaning, the samples were suitable for the application of protective coatings and for reuse. Sample weight loss close to 0.01% did not significantly affect the reusability. This is particularly important for semi-professional applications as well as for renovating hard-to-reach machine parts.

Scientific literature shows that the greater surface roughness of the metal parts of machines weakens the corrosion resistance due to the increase in the actual contact surface. The surface roughness parameters have a significant impact on the corrosion wear. After cleaning the spring samples, it was observed that the values of the highest *SP* elevations and *SV* grooves depths are in the range of 4.37 to 13.14 μm.

Obtained values of spatial surface roughness parameters such as *Sku* in the range of 9.58 to 35.57 μm and *Sq* in the range of 0.17 to 0.21 μm mean that the corrosion process has been weakened, which is positive/desirable and significantly extends the life span of machine parts.

Samples that were cleaned with basalt and glass are characterized by large depressions in the surface layers that facilitate the formation of corrosion micro-cells. This can be seen in Figure 13. Further evidence is provided by the increased values of the *S10z* parameter (see Table 3).

Finally, it was revealed that the best surface quality was obtained using fine gravel and the worst one using basalt. Each of the cleaning materials (abrasives) used during this research can be used to clean machine parts with complex or unusual shapes. However, the choice of abrasive significantly affects the micro-surface of the cleaned material. The test results discussed in the paper may be the basis for subsequent tests at changing process conditions. The rotational-abrasive method of cleaning machine parts used in this work is an alternative to chemical methods that are known and used on the market. At last, the “Corg ver. 1” device proved to be able to effectively clean corroded machine parts by means of the rotational method, with low economic effort.

## Figures and Tables

**Table 1:** Weight distribution among investigated sample groups.

Samples Group Number	Weight of the 1st Sample (g)	Weight of the 2nd Sample (g)	Weight of the 3rd Sample (g)	Weight of the 4th Sample (g)	Weight of the 5th Sample (g)
Group 1	18.64	18.70	18.60	18.67	18.88
Group 2	18.87	18.93	19.20	18.89	19.10
Group 3	18.68	18.56	18.45	18.77	18.96
Group 4	18.94	18.47	19.00	18.93	18.78
Group 5	18.87	19.10	18.56	19.12	18.45

**Table 2:** Weight loss distribution in groups of spring samples.

Samples Group Number	Abrasive Type	Average Weight of Samples in the Group; before Cleaning (g)	Average Weight of Samples in the Group; after Cleaning (g)	Average Sample Weight Loss in the Group (g)	Standard Deviation
Group 1	grinding stone	18.80	18.57	0.23	0.10
Group 2	basalt	18.75	18.56	0.19	0.14
Group 3	fine gravel	18.76	18.55	0.21	0.19
Group 4	glass	18.87	18.68	0.19	0.21
Group 5	sand	18.87	18.68	0.19	0.30

**Table 3:** Three-dimensional (3D) surface roughness parameters computed for the groups of spring samples.

Description	Name	Unit	Value					
			Group 1	Group 2	Group 3	Group 4	Group 5	Standard Deviation
Average height of selected area	*Sa*	μm	0.69	0.81	1.44	0.87	0.59	0.33
Root mean square height of selected area	*Sq*	μm	0.96	1.24	2.32	1.21	0.83	0.58
Maximum peak height of selected area	*Sp*	μm	19.38	8.32	12.74	20.54	14.45	4.99
Maximum valley depth of selected area	*Sv*	μm	10.83	21.46	28.68	8.66	10.08	8.74
Maximum height of selected area	*Sz*	μm	30.22	29.79	41.42	29.21	24.53	6.23
Ten-point height of selected area	*S10z*	μm	24.17	27.54	37.28	25.47	20.31	6.34
Skewness of selected area	*Ssk*	-	−0.92	−3.35	−3.41	0.05	−0.81	1.58
Kurtosis of selected area	*Sku*	-	13.03	35.57	26.52	10.74	9.58	11.44
Root mean square gradient of selected area	*Sdq*	-	0.17	0.19	0.21	0.21	0.17	0.02
Developed interfacial area ratio of selected area	*Sdr*	%	1.38	1.90	2.14	2.16	1.54	0.35
E Flatness using least squares reference plane of selected area	*FLTt*	μm	30.22	29.79	41.42	29.21	24.53	6.23
Lambda C: cutoff wavelength of selected area	*Lc*	μm	800	800	800	800	800	0

**Table 4:** The summary of microhardness measurement results in the HV scale.

Place of Measurement	Sample Number					
	1		2		3	
	A	B	A	B	A	B
1	401.5	482.7	440.2	564.8	579.7	544.5
	376.6	464.1	532.6	529.4	505.9	486.2
	391.1	499.1	409.3	568.2	529.7	432.8
	368.8	498.4	422.5	580.4	508.6	454.7
2	475.7	495.5	571.1	510.5	548.6	602.1
	472.1	488.9	514.8	508.6	523.3	554.6
	495.8	493.7	469.4	434.0	440.0	480.3
	429.9	526.4	493.0	492.7	459.0	612.9
3	361.6	525.5	414.2	560.1	547.4	458.8
	378.3	482.5	435.9	472.8	575.5	508.9
	392.2	502.8	465.0	413.8	525.0	471.1
	427.8	518.3	483.7	522.2	451.8	560.8
4	338.8	480.0	467.3	524.1	507.8	515.0
	459.5	529.7	445.4	550.1	563.9	565.1
	371.6	461.8	541.2	519.7	588.9	522.7
	353.3	440.0	478.8	581.0	594.3	486.9
5	346.0	442.1	503.6	550.1	564.8	600.7
	371.1	459.0	510.7	543.3	540.1	547.4
	358.5	480.0	489.9	560.8	466.2	502.5
	319.9	442.8	557.7	548.0	489.2	519.4
Average (HV)	394.51	485.67	482.32	526.73	525.49	521.37
Standard deviation (HV)	49.70	27.82	46.79	45.41	46.70	51.49

**Table 5:** Qualitative classification of abrasives used in the research process.

Rank	Abrasive Fraction (mm)	Abrasive Name
1 (the best)	0.1–0.4	fine gravel
2	0.1–10.0	grinding stone
3	0.1–15.0	glass
4	0.2–0.5	sand
5 (the worst)	0.2–0.8	basalt
